# How would Socrates debrief? Five tools using original Socratic dialogue

**DOI:** 10.12688/mep.21414.1

**Published:** 2026-03-10

**Authors:** Matthew Bowker, Amy Younger, Richard Thomson

**Affiliations:** 1 Newcastle University Faculty of Medical Sciences, Newcastle upon Tyne, England, UK; 2 University of Sunderland Faculty of Health Sciences and Wellbeing, Sunderland, England, UK; 3 Northumbria Healthcare NHS Foundation Trust, North Shields, England, UK

**Keywords:** Socratic questioning, healthcare simulation, simulation debriefing, facilitation orientations, productive uncertainty

## Abstract

Simulation facilitators routinely invoke the ‘Socratic method’ when describing their questioning approach, yet this invocation often lacks philosophical grounding and practical specificity. Whilst Socratic questioning features prominently in debriefing standards, its application has become what scholars describe as "extraordinarily vague", with conflicting interpretations proliferating across the literature. Facilitators need clear guidance for important decisions: when to challenge versus support, when to profess ignorance versus share expertise, when to create discomfort versus maintain psychological safety. This article returns to Plato’s dialogues to construct a contemporary pedagogical framework through close textual analysis. We developed five distinct facilitation orientations drawn from specific passages in the original texts: the Gadfly (challenging assumptions through persistent questioning), the Professed Ignorant (modelling intellectual humility), the Midwife (facilitating emergence of tacit knowledge), the Stingray (inducing productive cognitive dissonance), and the Co-inquirer (fostering collaborative discovery). These orientations function as philosophical stances rather than algorithmic techniques, providing meta-level guidance that complements existing debriefing frameworks. Each orientation addresses different aspects of productive uncertainty, the deliberate cultivation of intellectual discomfort as a catalyst for deeper thinking. When facilitators position themselves as fellow learners, debriefing can shift from teaching learners what to think towards teaching them how to think. Engagement with Socratic principles expands facilitators’ repertoires for creating meaningful learning conversations. These orientations offer simulation educators a philosophically grounded alternative to vague appeals to ‘being Socratic’. They emerge from interpretive choices calibrated specifically to healthcare simulation contexts rather than claims of historical authenticity.

## Introduction

Simulation facilitators frequently invoke the “Socratic method” when describing their questioning approach, yet this invocation often lacks philosophical grounding or practical clarity. While Socratic questioning features prominently in debriefing standards ^1^ its application in healthcare simulation has become what Fey *et al.* describe as “extraordinarily vague,” with “the literature rife with conflicting interpretations.” ^2(p4)^ Although attention has been given to perversions of the method such as ‘pimping’, little work has translated the original Platonic dialogues into practical frameworks for contemporary facilitators. Without such translation, we risk perpetuating questioning approaches that bear little resemblance to, or worse, actively contradict, the philosophical principles they claim to embody.

Our knowledge of the Socratic method comes primarily from Plato’s dialogues, texts rich in philosophical complexity that admit multiple interpretations. This presents both challenge and opportunity: while no single “authentic” Socratic method can be recovered from these ancient texts, engagement with the original sources can generate pedagogical insights unavailable through second-hand references alone. What remains unexplored is how specific passages from Plato’s dialogues might be translated into distinct facilitation orientations that address the practical challenges simulation educators face in debriefing conversations.

This gap matters because vague invocations of “being Socratic” provide insufficient guidance for the decisions facilitators must make: when to challenge versus support, when to profess ignorance versus share expertise, when to create discomfort versus maintain psychological safety. With debriefing quality fundamentally shaping learners’ development of clinical reasoning and reflective practice, we require frameworks that bridge philosophical principles and pragmatic action. Here, we offer such a bridge: five distinct facilitation orientations constructed from close reading of Plato’s dialogues, designed to help facilitators think more deliberately about the purpose and function of their questioning.

### The Gadfly

The metaphor of the “gadfly” appears in Plato’s *Apology*, where Socrates defends his philosophical mission before the Athenian court. In this compelling passage, Socrates characterises his role as one who awakens the complacent:

*“If you kill me, you will not easily find such another man as I, a man who – if I may put it a bit absurdly – has been fastened as it were to the City by the God as, so to speak, to a large and well-bred horse, a horse grown sluggish because of its size and in need of being roused by a kind of gadfly. Just so, I think, the God has fastened me to the City. I rouse you. I persuade you. I upbraid you. I never stop lighting on each one of you, everywhere, all day long.”* (Apology, 30e-31a) ^3^

This striking imagery reveals the essential purpose of the Socratic gadfly: to prevent complacency and intellectual stagnation. Socrates’ threefold action – to rouse, persuade, and upbraid – captures a persistent, purposeful challenge to unexamined thinking. The gadfly approach strategically disrupts comfortable assumptions, pressing learners to examine their reasoning more rigorously. For simulation educators, the gadfly stance involves strategically questioning learners’ premature certainty and challenging unexamined assumptions.

### Tips

When applied within debriefing, this approach might involve asking questions that:

• Challenge diagnostic momentum
• Question routine interventions that lack evidential support
• Examine the influence of cognitive biases on clinical decisions

For example, a facilitator might ask: “I noticed that once the diagnosis of sepsis was suggested, all team members accepted it despite contradictory findings. What led to this collective certainty?”

### The professed ignorant: Establishing intellectual humility

Socrates’ claim to know only that he does not know represents both personal humility and a deliberate teaching strategy. In the *Apology*, he articulates this position with clarity:

*“I do know that I am wise in this one small respect: I do not think I know what I do not.”* (Apology, 21d) ^3^

By positioning himself as a learner rather than an authority, Socrates creates an intellectual environment that empowers students to examine their own thinking without fear of judgement. This approach dismantles hierarchical structures that inhibit honest inquiry. As he elaborates:

*“For to fear death, Gentlemen, is nothing but to think one is wise when one is not; for it is to think one knows what one does not know.”* (Apology, 29a)

In clinical contexts, this insight has particular relevance. The clinician who cannot admit uncertainty may pursue harmful interventions rather than acknowledge the limits of their knowledge. By modelling comfort with not knowing, facilitators help future clinicians develop the intellectual courage to practice medicine with appropriate humility.

### Tips

Within debriefing, the Professed Ignorant stance might involve:

• Explicitly acknowledging the limits of one’s own expertise
• Approaching the learner’s thinking with genuine curiosity
• Avoiding leading questions that fish for predetermined answers

## The Midwife: Facilitating knowledge emergence

Socrates explicitly compares his teaching method to midwifery in Plato’s *Theaetetus*, where he describes his role not as one who imparts wisdom but as one who helps others deliver insights already within them:

*“I am so far like the midwife that I cannot myself give birth to wisdom, and the common reproach is true, that, though I question others, I can myself bring nothing to light because there is no wisdom in me. The reason is this. Heaven constrains me to serve as a midwife, but has debarred me from giving birth… The many admirable truths they bring to birth have been discovered by themselves from within. But the delivery is heaven’s work and mine.”* (Theaetetus, 149b-150c) ^4^

This compelling metaphor highlights Socrates’ understanding of his pedagogical role – not as one who imparts wisdom, but who assists others in articulating what they already tacitly know. Through questioning, he helps bring forth hidden knowledge, just as a midwife assists in birth.

### Tips

In debriefing, the midwife stance involves facilitating the emergence of knowledge through structured questioning that:

• Draws connections between disparate elements of understanding
• Builds from known to unknown concepts through scaffolding questions
• Positions the facilitator as guide rather than source of wisdom

For instance, a facilitator might ask: “Before we discuss your management of the patient, can you identify what patterns you noticed in the patient’s initial presentation that informed your approach?”

## The stingray: Inducing cognitive reframing

Perhaps the most provocative aspect of the Socratic method is what scholars have termed the “stingray” approach. In the *Meno*, the protagonist compares Socrates to a torpedo fish that numbs upon contact:

*“You are […] like the stingray in the sea, which benumbs whatever it touches. I think you’ve now done something of the sort to me. My tongue, my soul, are numb – truly – and I cannot answer you.”* (Meno, 80a-b) ^3^ ^(p162)^

This metaphor captures how Socratic questioning can induce a form of productive disorientation – a state where existing knowledge frameworks are temporarily destabilised to create space for new understanding. Euthyphro describes a similar sensation:

*“But, Socrates, I do not know how to tell you what I mean. Somehow everything I propose goes round in circles on us and will not stand still.”* (Euthyphro, 11b) ^3^ ^(p52)^

This state of aporia – perplexity or puzzlement – represents not failure but progress. It marks the moment when a learner recognises the inadequacy of their current understanding and becomes receptive to deeper exploration.

### Tips

In debriefing, the stingray stance involves deliberately inducing cognitive dissonance through:

• Presenting counterfactual scenarios (“What if the patient had been…”)
• Challenging mental models by introducing unexpected variables
• Asking “what if” questions that require conceptual reframing
• Creating moments of productive confusion that stimulate new thinking

## The Co-inquirer

A central tenet of the Socratic dialogues is the sense of co-discovery and camaraderie; while the teacher-student relationship is present, it exists within a framework of humility and mutual high regard. Socrates does not position himself as the final arbiter of truth and knowledge. Rather, he role-models his own experience of discovery and fallibility and invites his students to support him on this journey.

This is beautifully illustrated as Socrates is about to cross-examine claims made by Callicles:

*“I am convinced that if you agree with the opinions held by my soul, then at last we have attained the actual truth. For I observe that anyone who is to test adequately a human soul for good or evil living must possess three qualifications, all of which you possess, namely knowledge, good will, and frankness. Now I encounter many who cannot test me because they are […] unwilling to tell the truth because they do not care for me as you do.”* (Gorgias, 486e) ^5^

*“As for me, if I act wrongly at all in the conduct of my life, you may be assured that my error is not voluntary but due to my ignorance. Now that you have begun to admonish me, therefore, do not give it up, but reveal to me clearly what course I must follow and how I may achieve it.”* (Gorgias, 488a) ^5^

The stance of ‘co-inquirer’ is perhaps the form of Socratic inquiry which is furthest removed from pimping. Pimping – often confused with the Socratic method – is characterised by unidirectional inquiry with little recourse for the student to probe their teacher’s beliefs. ^6^ Co-inquiry, by contrast, positions the facilitator and learner as fellow seekers within a reciprocal dialogue. Rather than testing the learner’s knowledge through pointed questioning, the co-inquirer joins them in genuine exploration, modelling uncertainty and intellectual curiosity. The facilitator’s questions arise from authentic interest rather than hidden knowledge, and the learner’s responses shape the direction of inquiry. This reciprocity creates what might be termed a ‘joint inquiry’ – an investigation where both parties contribute observations, test hypotheses together, and remain open to surprise.

### Tips

How might we enact this stance of co-inquiry within clinical debriefing? The challenge lies in translating this philosophical position into concrete conversational moves. Several practical strategies can help facilitators embody the role of fellow seeker:

• Frame the debrief as a joint investigation: Adopt genuine curiosity with questions like, “Help me understand your perspective,” positioning the learner as an expert on their experience.
• Establish psychological safety through mutual respect: State that the purpose is shared learning, not judgment. Assume learners acted with good intent, creating a safe space for honest reflection.
• Role-model fallibility to build camaraderie: Lower the hierarchy by admitting your own uncertainties or sharing relevant past mistakes, which builds trust and encourages openness.
• Focus on ‘frames’ not just ‘facts’: Prioritise exploring the learner’s thought process (their ‘frame’) over simply listing correct or incorrect actions to jointly understand the reasoning.
• Explicitly invite challenge and correction: Empower learners by asking, “Does that match your experience?” to ensure the final understanding is shared and accurate.

## Discussion

Our analysis is a construction of a contemporary pedagogical framework inspired by ancient texts. The five orientations we have developed represent interpretive choices rather than direct translations, acknowledging what Reich describes as the fundamental challenge of discerning any singular method from Socrates’ varied approaches across different dialogues. ^7^ This interpretive dimension illuminates how simulation educators might think differently about questioning, uncertainty, and the facilitator’s role in debriefing conversations.

### The constructed nature of Socratic practice

Scholarly literature reveals significant debate about whether Socrates possesses a coherent method at all. ^8^ Reich’s analysis demonstrates the paradoxical nature of Socratic discourse, where “utter certainty and utter ignorance” appear simultaneously, raising questions about whether *docta ignorantia* represents genuine humility or performance. ^7^ Rather than claiming to have discovered timeless techniques, we position our orientations as contemporary constructions that draw selectively from Platonic texts to address specific challenges in simulation debriefing.

This constructed nature aligns with broader patterns in how disciplines adapt Socratic principles. Dinkins and Cangelosi’s work in nursing education demonstrates similar interpretive processes, whilst Kessels’ application to organisational learning reveals how different professional contexts generate distinct Socratic constructions. ^9, 10^ Our contribution lies in developing orientations specifically calibrated to healthcare simulation debriefing.

### From translation to orientation

Rather than offering algorithmic techniques, our five orientations function as interactional mindsets – ways of approaching debriefing conversations that reflect certain Socratic sensibilities without claiming direct correspondence to ancient practice. This shift acknowledges what discursive psychology reveals about the contextual nature of interactional practices. ^11^ The same question can function differently across different conversational contexts - challenging assumptions effectively in one situation may appear aggressive or inappropriate in another, depending on relationships, timing, and local dynamics. Our orientations provide philosophical frameworks for thinking about local choices rather than prescriptive formulas for conducting them.

### The pedagogy of productive uncertainty

The five orientations converge around productive uncertainty – deliberate cultivation of intellectual discomfort as a catalyst for deeper thinking. When *Meno* describes feeling “numb” and unable to answer, this represents a threshold moment where existing knowledge frameworks prove inadequate and new learning becomes possible. However, inducing such moments in simulation requires considerable skill in distinguishing between productive confusion that stimulates thinking and destructive anxiety that inhibits learning.

The Co-inquirer orientation provides important guidance by positioning facilitators as fellow seekers rather than judges, maintaining psychological safety whilst creating intellectual tension necessary for growth. This represents a departure from hierarchical models towards collaborative approaches that recognise learning as mutual discovery.

### Rethinking facilitator identity

Implementing these orientations requires shifts in how facilitators understand their role. The Professed Ignorant orientation challenges perceptions that admitting uncertainty can be perceived as weakness rather than pedagogical strength. The tension between modelling humility and maintaining educational authority requires careful navigation, particularly given patient safety concerns that create legitimate expectations of clinical competence.

### Positioning the framework

Simulation debriefing benefits from substantial scholarship on effective practice. Numerous frameworks provide structure for debriefing conversations, conversational strategies guide the analysis phase, and specific techniques like advocacy-inquiry help facilitators explore learners’ reasoning with “good judgement”. ^12^ This body of work has generated consensus around core principles: the importance of psychological safety, the value of structured approaches, and the centrality of reflection to learning. ^13^

Our contribution operates at a different level. Where existing scholarship addresses how to debrief -providing structures, techniques, and conversational strategies - we address who the facilitator is being during these conversations. The five orientations function as philosophical stances that inform moment-to-moment decisions: when to challenge versus support, when to claim ignorance versus share expertise, when to induce discomfort versus maintain safety.

Consider a facilitator using advocacy-inquiry to explore a learner’s clinical reasoning. The same technique functions differently depending on whether the facilitator adopts a Gadfly stance (challenging unexamined assumptions), a Midwife stance (helping articulate tacit knowledge), or a Co-inquirer stance (genuinely uncertain about the best approach). The orientations thus complement rather than replace existing frameworks, offering a meta-level consideration of facilitation identity that can inform which techniques to deploy and when.

### Navigating cultural contexts

The five orientations we propose emerge from Western philosophical traditions and carry implicit cultural assumptions about learning relationships. Most notably, several orientations - particularly the Gadfly and Stingray - endorse intellectual challenge and productive discomfort in ways that may conflict with learning cultures where questioning authority figures violates established social norms.

Langen and Stamov Roßnagel’s research demonstrates that Socratic questioning creates measurably higher stress for learners from Confucian heritage cultures compared to their Western peers. ^14^ This finding carries practical implications for facilitators working in culturally diverse teams. The Co-inquirer orientation may offer a culturally responsive starting point, as its emphasis on mutual respect and collaborative discovery aligns more closely with relational approaches to learning. Facilitators might then gauge learners’ receptiveness to more challenging stances like the Gadfly, recognising that what constitutes “productive” discomfort varies across cultural contexts.

This cultural specificity does not invalidate these orientations but reminds us that all pedagogical frameworks reflect particular cultural values. Facilitators bear responsibility for adapting their approach to consider learners’ cultural backgrounds while still creating conditions for meaningful learning. Future work might explore how these orientations translate across diverse cultural contexts in healthcare education.

### Limitations and future directions

Our interpretive approach creates several important limitations. The selective nature of our textual analysis means other readings of Plato’s dialogues could yield different pedagogical insights. The abstract nature of our orientations creates additional challenges for practical implementation. Future research might examine how these orientations function in actual debriefing conversations, attending to the local interactional work required to implement them effectively.

### Implications for practice

Rather than treating these orientations as techniques to be applied, we suggest they function as philosophical resources for thinking about the facilitator’s role and purpose. The value lies not in faithful reproduction of these methods but in how engagement with Socratic principles might expand facilitators’ repertoires for creating meaningful learning conversations.

This represents a move from technical rationality towards reflective practice in debriefing facilitation. Rather than seeking optimal techniques for achieving predetermined outcomes, these orientations invite facilitators to think more philosophically about the nature of learning, the value of uncertainty, and the possibilities inherent in genuine dialogue.

## Conclusion

Our exploration reveals both possibilities and challenges in adapting ancient philosophical practices for contemporary healthcare education. Rather than claiming historical authenticity, we have developed interpretive frameworks that draw selectively from Platonic texts to address specific challenges in debriefing practice.

### Practicing what we preach

As authors who have just urged you toward intellectual humility, we must model it ourselves. We leave you with the question that troubles our own practice: **In your last debriefing, were you teaching your learners to think, or were you teaching them to think like you?**

If this question creates discomfort, we suggest that’s exactly where learning begins.

## Data Availability

No new data were used in this manuscript.
