# Supplementary material for: How would Socrates debrief? Five tools using original Socratic dialogue
Source: MedEdPublish (2016). 2026 Mar 10;16:5. [Version 1] doi: 10.12688/mep.21414.1 (PMC13032099; doi:10.12688/mep.21414.1)
Supplement: Peer review history

# Reviewer report: Reviewer response for version 1

**DOI:** 10.21956/mep.22935.r45587

**Published:** 2026-03-27

**Authors:** Mary Fey

**Affiliations:** 1 Harvard Center for Medical Simulation, Boston, USA

This article offers a novel and compelling framework for considering the range of "stances" a simulation facilitator may adopt during debriefing. As our understanding of debriefing has evolved, it has become increasingly clear that no single approach serves all learners or contexts. This article provides a fresh perspective that illuminates the nuanced repertoire of skills that proficient debriefers draw upon to meet the needs of diverse learners in complex learning environments where productive dialogue serves as the primary teaching and learning strategy.

 Each section offers a contemporary interpretation of Plato's dialogues to help the reader understand what Socrates sought to achieve through various approaches with his students. These interpretations are followed by practical, accessible examples of what a modern-day Socrates — that is, a skilled debriefer — might do to enact each strategy.

 There is one area I would encourage the authors to reexamine. The first concerns psychological safety. The authors suggest that the Stingray, who introduces cognitive dissonance, makes a choice to "create discomfort versus maintain psychological safety." This framing is at odds with Edmondson's conception of the construct. Edmondson defines psychological safety as the belief that a team is safe for interpersonal risk-taking — that individuals will not be shamed or humiliated for speaking up, asking questions, or admitting ignorance (1999, 2019). In the simulation context specifically, Rudolph et al. articulate that psychological safety creates a space in which learners feel *safe enough to embrace discomfort* in the service of learning (2014). Discomfort and psychological safety are therefore not opposing forces; rather, psychological safety is the very condition that makes productive discomfort possible. It follows, then, that the Stingray — perhaps more than any other facilitator stance — bears the greatest responsibility for actively establishing and maintaining psychological safety, so that learners are genuinely positioned to benefit from a well-aimed sting.

 References;

 Edmondson, A. C. (2019). The Fearless Organization. John Wiley & Sons.

If evidence from practice is presented, are all the underlying source data available to ensure full reproducibility?

Not applicable

Is the topic of the practical tips discussed accurately in the context of the current literature

Yes

Are all factual statements correct and adequately supported by citations?

Yes

Are the conclusions drawn balanced and justified on the basis of the presented arguments?

Yes

Are arguments sufficiently supported by evidence from the published literature and/or the authors’ practice?

Yes

Reviewer Expertise:

Debriefing, simulation

I confirm that I have read this submission and believe that I have an appropriate level of expertise to confirm that it is of an acceptable scientific standard.

## References

[1] : Establishing a Safe Container for Learning in Simulation. *Simulation in Healthcare: The Journal of the Society for Simulation in Healthcare* .2014;9(6) : 10.1097/SIH.0000000000000047 339-349 10.1097/SIH.0000000000000047 25188485

[2] : Psychological Safety and Learning Behavior in Work Teams. *Administrative Science Quarterly* .1999;44(2) : 10.2307/2666999 350-383 10.2307/2666999

# Reviewer report: Reviewer response for version 1

**DOI:** 10.21956/mep.22935.r45538

**Published:** 2026-03-25

**Authors:** Nathan Oliver

**Affiliations:** 1 University of Canberra, Canberra, Australia

Thank for such a thoughtful and insight generating paper, I sincerely enjoyed the read. 

 The authors address the problem, gap, and hook of the paper with clarity. Whilst I found myself at the beginning of the article wondering whether it matters if debriefers draw from the ancient texts of Plato within our contemporary debriefing, the authors were able to name 'Socratic method' within the INACSL standards and draw in scholarly debate on the matter. In the end for me, I wasn't convinced one way or another as to whether it *should *be listed as an approach within the standards, I was certainly convinced of the wisdom it has to offer us in real time debriefing contexts - as explored in the paper. 

 A few, more specific thoughts from me as I review the paper:

 - With the 'Gadfly' section, the authors offer a tip 'Challenge diagnostic momentum'. I think I know what meant here, a warning about rushing too quickly to a diagnosis without considering all elements, yet I wonder if it might be made more clear?

 - I really enjoyed the section the Professed Ignorant. A paper that might add to this point is: Molloy E, Bearman M. Embracing the tension between vulnerability and credibility: 'intellectual candour' in health professions education. Med Educ. 2019 Jan;53(1):32-41. doi: 10.1111/medu.13649. Epub 2018 Sep 7. PMID: 30192024.

 - Additional point, I noticed some symmetry across the paper with examples offered to help the reader see how these orientations might play out in a debrief. I would love to see one in the Professed Ignorant or Stingray sections. 

 - The Midwife heading seems far larger in the text here than previous or later points. I'm not sure this is deliberate but presume not?

 - The discussion was excellent and worked to take the examples given and give them a home in contemporary debriefing. The authors were careful to position these as orientations that sit above the technique or structure. 

 - I loved the final question at the conclusion.

If evidence from practice is presented, are all the underlying source data available to ensure full reproducibility?

Not applicable

Is the topic of the practical tips discussed accurately in the context of the current literature

Yes

Are all factual statements correct and adequately supported by citations?

Yes

Are the conclusions drawn balanced and justified on the basis of the presented arguments?

Yes

Are arguments sufficiently supported by evidence from the published literature and/or the authors’ practice?

Yes

Reviewer Expertise:

Simulation Debriefing, Simulation-Based Education, Translational Simulation

I confirm that I have read this submission and believe that I have an appropriate level of expertise to confirm that it is of an acceptable scientific standard.
